# Ebola virus infection kinetics in chimeric mice reveal a key role of T cells as barriers for virus dissemination

**DOI:** 10.1038/srep43776

**Published:** 2017-03-03

**Authors:** Anja Lüdtke, Paula Ruibal, David M. Wozniak, Elisa Pallasch, Stephanie Wurr, Sabrina Bockholt, Sergio Gómez-Medina, Xiangguo Qiu, Gary P. Kobinger, Estefanía Rodríguez, Stephan Günther, Susanne Krasemann, Juliana Idoyaga, Lisa Oestereich, César Muñoz-Fontela

**Affiliations:** 1 Heinrich Pette Institute, Leibniz Institute For Experimental Virology, Martinistrasse 52 20251 Hamburg, Germany; 2 Department of Virology, Bernhard Nocht Institute for Tropical Medicine, 20359 Hamburg, Germany; 3 German Center for Infection Research (DZIF), Partner site Hamburg, Germany.; 4 Special Pathogens Program, National Microbiology Laboratory, Public Health Agency of Canada, Winnipeg, MB R3E 3R2, Canada; 5 Department of Microbiology, Immunology & Infectious Diseases, Université Laval, Quebec City, Canada; 6 Institute for Neuropathology, University Medical Center Hamburg-Eppendorf, 20251 Hamburg, Germany; 7 Department of Microbiology and Immunology, Stanford University School of Medicine, Stanford, CA 94305, USA

## Abstract

Ebola virus (EBOV) causes severe systemic disease in humans and non-human primates characterized by high levels of viremia and virus titers in peripheral organs. The natural portals of virus entry are the mucosal surfaces and the skin where macrophages and dendritic cells (DCs) are primary EBOV targets. Due to the migratory properties of DCs, EBOV infection of these cells has been proposed as a necessary step for virus dissemination via draining lymph nodes and blood. Here we utilize chimeric mice with competent hematopoietic-driven immunity, to show that EBOV primarily infects CD11b^+^ DCs in non-lymphoid and lymphoid tissues, but spares the main cross-presenting CD103^+^ DC subset. Furthermore, depletion of CD8 and CD4 T cells resulted in loss of early control of virus replication, viremia and fatal Ebola virus disease (EVD). Thus, our findings point out at T cell function as a key determinant of EVD progress and outcome.

EBOV is a negative-stranded RNA virus, which belongs to the *Filoviridae* family and causes severe systemic disease in humans and non-human primates (NHP) with high case-fatality ratios. Epidemiology studies indicate that direct contact with infected body fluids is the main mode of transmission between humans^1^,^2^, which points out at the skin and the mucosal surfaces as main portals of EBOV entry^2^. Previous studies have demonstrated replication of EBOV in macrophages and dendritic-like cells in NHP tissue sections early after infection^3^,^4^, and suggested that macrophages and DCs were early virus targets. The notion that DC infection is an important event for EBOV pathogenesis has been further substantiated by the finding that EBOV infection impairs DC function *in vitro*^5^,^6^. Based on these previous findings and due to the migratory properties of DCs, the current hypothesis is that EBOV infection of DCs at the portals of virus entry leads to virus dissemination through the lymphatic system and blood^7^,^8^. However, this hypothesis has been difficult to test *in vivo* due to the lack of small animal models of EBOV infection. Inbred laboratory mice are completely resistant to infection with filoviruses and only mouse models with different degrees of immunosuppression are susceptible to infection with non-adapted EBOV^9^. This lack of immunocompetent animal models has precluded endpoint studies to elucidate the kinetics of EBOV infection *in vivo*.

The last decade of research has generated important advances in our knowledge of DC biology. It is now clear that DCs comprise many subsets with overlapping and non-overlapping functions and with different ontogeny^10^. Classic DCs are characterized by their dependence on FMS-like tyrosine kinase 3 (Flt3) and its ligand and are formed by several subsets in peripheral as well as lymphoid tissues. Plasmacytoid DCs are also dependent on Flt3 but are morphologically and functionally distinct. Finally, inflammatory DCs are derived from activated monocytes that infiltrate tissues during inflammation or infection^11^. Thus, previous *in vitro* experiments of EBOV infection of monocyte-derived DCs do not reflect the variety of DC subsets in living organisms. Currently, it is not known whether EBOV is equally capable of infecting all DC subsets *in vivo*.

Here we have investigated the kinetics of EBOV infection using chimeric mice that retained fully competent hematopoiesis. Upon mucosal exposure, EBOV productively infected resident macrophages and CD11b^+^ DCs in peripheral tissues and draining lymph nodes. Conversely, monocytes, neutrophils and cross-presenting tissue-resident CD103^+^ DCs were spared from infection. Inflammatory (monocyte-derived) CD11b^+^ DCs were preferred virus target cells at the onset of inflammation, after which both moDCs and conventional CD11b^+^ DCs supported EBOV infection. CD11b^+^ cells showed evidence of infection at both the mucosa and the draining lymph nodes. Depletion of T cells resulted in loss of control of local viral replication, virus dissemination and fatal Ebola virus disease (EVD). Our results identified infected CD11b^+^ DCs as putative viral vessels and underscored a key role of T cells as controllers of EBOV infection at the initial points of virus entry.

## Results

### Hematopoietic immune competence is required to control EBOV dissemination

**Figure 1:**
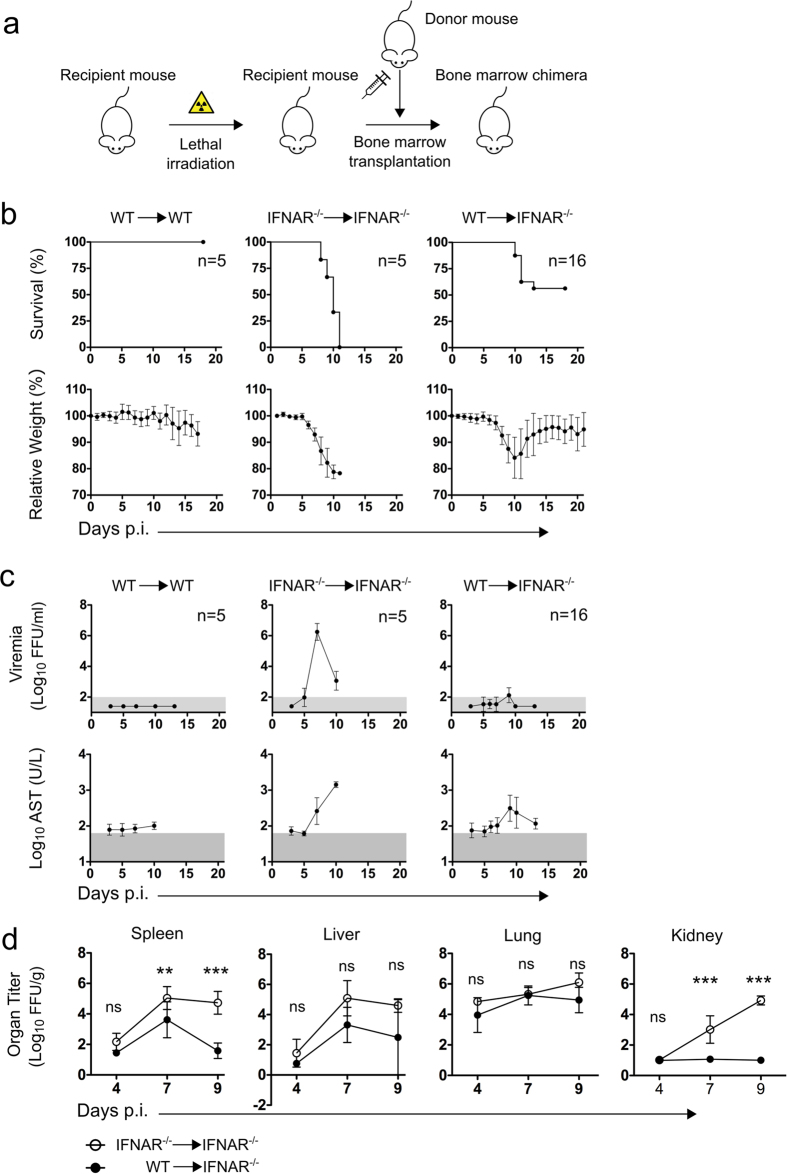
Chimeric mice indicate a role of hematopoietic cells on EBOV dissemination. Schematic of the generation of a bone marrow chimera (**a**). Four weeks post transplantation chimeric WT → WT mice, IFNAR^−/−^ → IFNAR^−/−^ mice and WT → IFNAR^−/−^ mice were infected i.n. with 1000 FFU of EBOV. Mice were monitored for survival and relative weight loss (**b**) and viremia in blood and AST activity were measured at indicated time points (**c**). Viral replication in spleen, liver, lung and kidney was determined at days 4, 7 and 9 post-infection. Statistical analysis was performed via non-parametric Kruskal-Wallis test followed by Dunn’s post-test. ns (not significant) when *p* > 0.05, *(*p* ≤ 0.05), **(*p* ≤ 0.01) and ***(*p* ≤ 0.001) (**d**). The normal range for AST and the limit of detection for viremia in blood are shaded in grey. Graphs represent mean value ± SD.

Previous studies have highlighted the important role of type I interferon (IFN-I) on host resistance to EBOV infection, particularly in the mouse model^9^,^12^. Consistently, IFN-I receptor knockout mice (IFNAR^−/−^) are susceptible to non-adapted EBOV via several routes. To study the kinetics of EBOV infection in an immune competent environment we generated bone marrow chimeras via whole body irradiation of IFNAR^−/−^ mice and transplantation of WT bone marrow progenitor cells from C57BL/6 donor mice. In these mice, hereafter referred to as WT → IFNAR^−/−^, IFN-I deficiency is restricted to the radio-resistant compartment (Fig. 1a). We hypothesized that partial IFN-I deficiency would allow EBOV replication to proceed and cause disease after which the hematopoietic-driven adaptive immune response would be essentially WT as we previously described for Lassa virus infection^13^. In fact, WT → IFNAR^−/−^ chimeras were susceptible to mucosal (intranasal) EBOV infection, displayed significant weight loss and survival rates of around 50% (Fig. 1b). WT → WT control chimeras were entirely resistant to infection while IFNAR^−/−^ → IFNAR^−/−^ succumbed to EBOV within the first 10 days after infection. These controls reflected the features of WT and IFNAR^−/−^ mice respectively and indicated that the results observed were not due to transplantation (Fig. 1b). Interestingly, WT → IFNAR^−/−^ chimeras did not show viremia (Fig. 1c) and had significantly lower virus titers in most peripheral organs compared to IFNAR^−/−^ → IFNAR^−/−^ control chimeras (Fig. 1d). These results strongly suggested that immune competence in the hematopoietic compartment was required to prevent virus dissemination from the initial point of virus entry.

### Kinetics of EBOV infection *in vivo*

**Figure 2:**
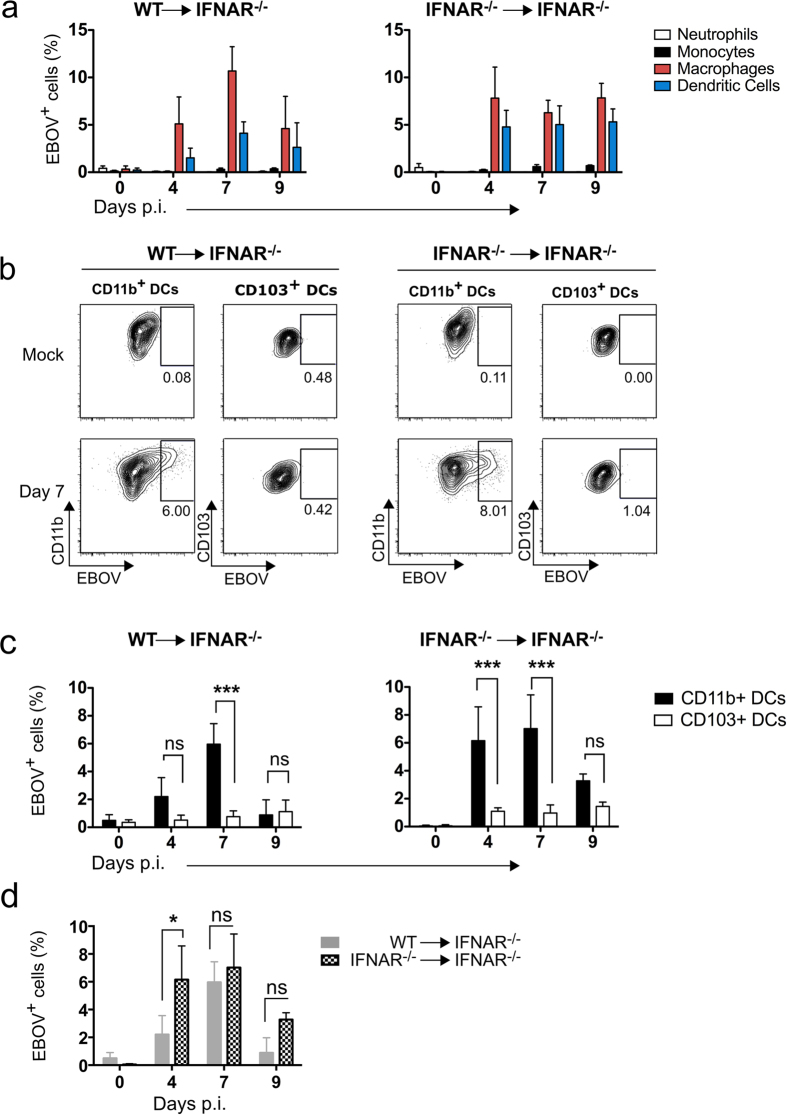
CD11b^+^, but not CD103^+^ dendritic cell subsets are infected during EVD infection. Chimeric WT → IFNAR^−/−^ mice and IFNAR^−/−^ → IFNAR^−/−^ mice were infected i.n. with 1000 FFU of EBOV. The infection of myeloid cells in lung was analyzed for *n* = 3 mice at days 4, 7 and 9 post infection using flow cytometry (**a**). Infected cells were identified using mononclonal anti-GP antibodies. Representative plots (**b**) and graphs (**c**) show surface staining of EBOV GP in CD11b^+^ and CD103^+^ DCs. Percentages of EBOV^+^ cells is presented as mean ± SD. Kinetics of infection showing differences in the frequency of infected cells in WT → IFNAR^−/−^ mice vs IFNAR^−/−^ → IFNAR^−/−^ chimeras (**d**). Across the figure statistical analyses were performed via non-parametric Kruskal-Wallis test followed by Dunn’s post-test. ns (not significant) when *p* > 0.05, *(*p* ≤ 0.05), **(*p* ≤ 0.01) and ***(*p* ≤ 0.001).

Our results indicated that loss of IFN-I responsiveness in the hematopoietic compartment resulted in virus dissemination suggesting a putative correlation between infection of IFN-deficient cells and virus spread in our model. To test this hypothesis we sought to determine the kinetics of EBOV infection after intranasal virus inoculation of WT → IFNAR^−/−^ chimeras as well as IFNAR^−/−^ → IFNAR^−/−^ mice. To track infected cells *in vivo* we utilized Alexa Fluor 488-conjugated anti-EBOV glycoprotein (GP) antibodies (clones 5D2 and 5E6)^14^ in combination with multiparametric flow cytometry (Supplementary Fig. S1). This strategy allowed identification of immune cell subsets productively infected with EBOV via detection of EBOV GP in the cell surface. Serial flow cytometric analysis of lung tissue from infected mice revealed that infection of alveolar resident macrophages and DCs were detectable via anti-GP staining at day 4 post-infection and was observable in both chimeras until the humane endpoint for IFNAR^−/−^ → IFNAR^−/−^ (day 9). Strikingly, the pattern of infection was not dependent on IFN-I competence but was restricted to DCs and macrophages (Fig. 2a). We did not detect expression of surface GP in other leukocyte populations such as neutrophils, monocytes, T cells and B cells, as well as in CD45^−^ stromal cells. These findings suggested that these cell subsets were not infected productively with EBOV, even though it is possible that they support levels of viral replication below the detection limit of our technique (Fig. 2a and Supplementary Fig. S2).

Tissue-resident DCs are composed at least by two subsets. CD103^+^ DCs express the C-type lectin langerin and play a major role cross-presenting viral antigens to CD8 T cells^15^. Conversely, CD11b^+^ DCs express DC-specific ICAM-3-grabbing non-integrin (DC-SIGN) and are heterogeneous, in particular during inflammation where this subset is formed by resident DCs as well as CD11b^+^ DCs derived from inflammatory monocytes^16^. In order to further determine the nature of EBOV-infected cells *in vivo*, we next sought to characterize the phenotype of virus-infected DCs. Flow cytometry analysis of infected DC populations allowed detection of surface EBOV GP in CD11b^+^ DCs but not in CD103^+^ DCs strongly suggesting that these cells were spared from infection, even though other possibilities such as non-productive infection cannot be ruled out (Fig. 2b). In any case, productive EBOV infection was restricted to the CD11b^+^ compartment perhaps reflecting the important role of DC-SIGN as cell attachment factor for EBOV^17^. Although the kinetics of infection was similar in WT → IFNAR^−/−^ and IFNAR^−/−^ → IFNAR^−/−^chimeras, the frequency of infected CD11b^+^ DCs was higher in the total absence of IFN-I competence at early time points after infection (day 4) (Fig. 2c and d). These results indicated that, while IFN-I did not influence the ability of EBOV to infect target cells, it restricted early viral replication.

**Figure 3:**
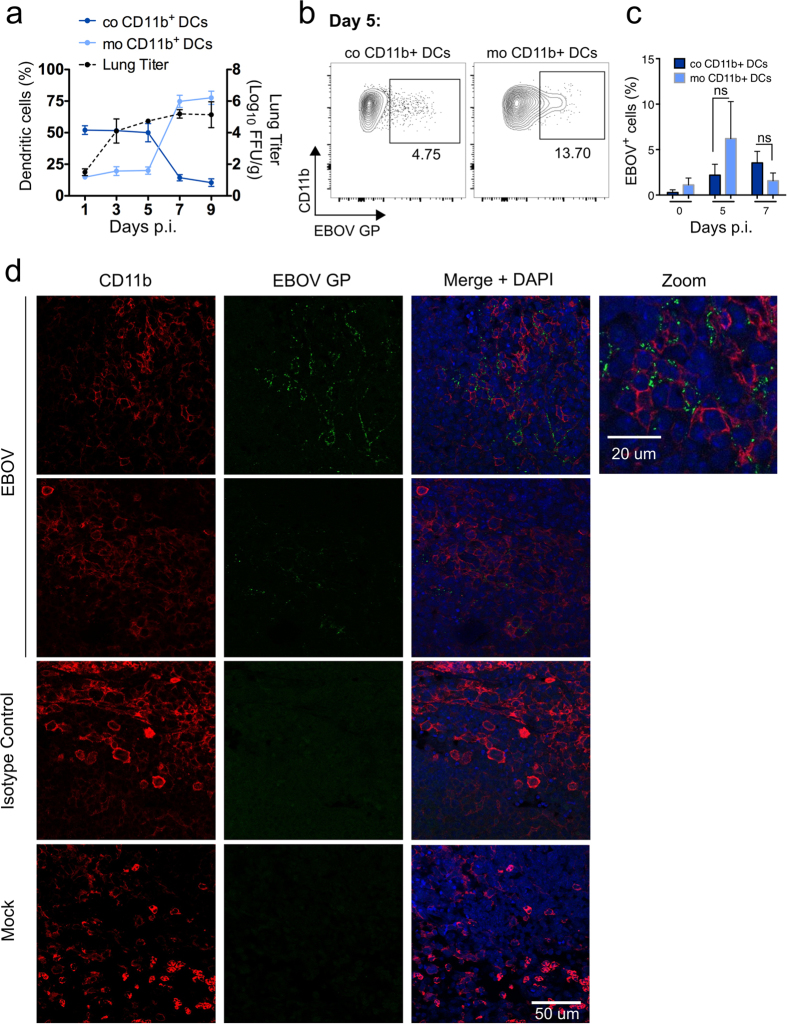
Infection kinetics of monocyte-derived and conventional CD11b^+^ DC subsets in lung and lymph nodes. IFNAR^−/−^ mice were infected i.n. with 1000 FFU of EBOV and 3 mice each were euthanized at days 1, 3, 5, 7 and 9 post infection. Frequencies of monocyte-derived CD11b^+^ DCs (moCD11b^+^ DCs) and conventional lung resident CD11b^+^ DCs (coCD11b^+^ DCs) within the dendritic cell population of the lung as well as lung titers were analyzed at indicated time points (**a**). CD11b^+^ DCs were gated as described in Supplementary Fig. S1. Discrimination of moCD11b^+^ DCs and coCD11b^+^ DCs was achieved using the markers MAR-1 and CD64. EBOV infection of moCD11b^+^ DCs and coCD11b^+^ DCs was determined using monoclonal anti-GP antibodies. Representative plots of infected CD11b^+^ DC subsets at day 5 are depicted in (**b**). Kinetics of infection of both DC subsets over time. Numbers represent the frequencies of infected cells within either co or moCD11b^+^ DCs. Statistics was assessed via Kruskal-Wallis test followed by Dunn’s post-test. ns (not significant) when *p* > 0.05, *(*p* ≤ 0.05), **(*p* ≤ 0.01) and ***(*p* ≤ 0.001). (**c**) Mediastinal lymph node section of an EBOV infected IFNAR−/− mouse was stained with AF488-conjugated mononclonal anti-GP antibodies and anti-CD11b antibody followed by a AF555-conjugated secondary anti-rabbit antibody. Two individual stainings of the same lymph node harvested 11 days post-infection are shown as well as staining of EBOV infected lymph nodes with AF488-conjugated isotype control antibody and staining of a mock mouse with AF488-conjugated mononclonal anti-GP antibodies (**d**).

Due to the fact that the CD11b^+^ compartment is heterogeneous, we next sought to identify the nature of EBOV-infected cells within this population. Staining of cells with CD64 and MAR-1 allowed specific discrimination between inflammatory monocyte-derived DCs (MAR-1^+^ CD64^+^) and conventional resident CD11b^+^ DCs (MAR-1^low^ CD64^−^) as previously described^18^ (Supplementary Fig. S1). As shown for other models of acute inflammation, the peak of EBOV replication coincided with the detection of high numbers of moDCs in the lung, which outnumbered resident CD11b^+^ DCs within the CD11b^+^ cell compartment at days 7–9 post-infection (Fig. 3a). At earlier time points (day 5) where the frequency of conventional CD11b^+^ DCs was significantly higher than that of moDCs (50% and 20% respectively) EBOV infection was detected to a higher extent in moDCs, suggesting that these cells were preferred early EBOV targets (Fig. 3a–c and Supplementary Fig. S3). Staining of tissue sections of mediastinal lymph nodes (mLNs) at the experimental endpoint (day 11) also revealed infection of CD11b^+^ cells (Fig. 3c). These results suggest that infected cells in the lungs may carry infectious virus to the mLNs or that infection of lymphoid CD11b^+^ cells, for example due to passive drainage of infectious EBOV from the lung, also serves to amplify EBOV infection outside the initial replication site.

Taken together, our data suggested that productive EBOV infection was restricted to the CD11b^+^ compartment and that infiltration of inflammatory monocytes and their differentiation into moDCs provided early viral targets that resulted in virus amplification. These findings are consistent with monocytes being refractory to EBOV infection and permissive only after their differentiation into moDCs^19^.

### T cells are required to prevent EBOV dissemination

EVD is a systemic disease and the levels of viremia are strongly correlated with fatal outcome. Thus, we sought to elucidate the mechanisms by which EBOV disseminates from the initial infection site to the body. Our results pointed out at inflammatory moDCs as important EBOV targets at the mucosa, and suggested that cross-presenting CD103^+^ DCs could retain T cell priming capacity during infection. Therefore, we specifically addressed the role of moDCs and T cells on EBOV dissemination via specific depletion of these cell subsets *in vivo*. To block monocyte infiltration into the respiratory tract upon infection we engineered chimeric mice in which hematopoietic cells harbored mutations in the C-C chemokine receptor type 2 (CCR2). CCR2 is required for efficient egress of monocytes from the bone marrow^20^ and for infiltration of monocytes into peripheral tissues during inflammation^21^. Perhaps surprisingly, blockade of monocyte infiltration did not result in reduction of EBOV replication in the lung (Supplementary Fig. S4). These findings suggested that while moDCs are EBOV targets, other cells in the respiratory tract such as alveolar macrophages and resident CD11b^+^ DCs were sufficient to support EBOV replication.

**Figure 4:**
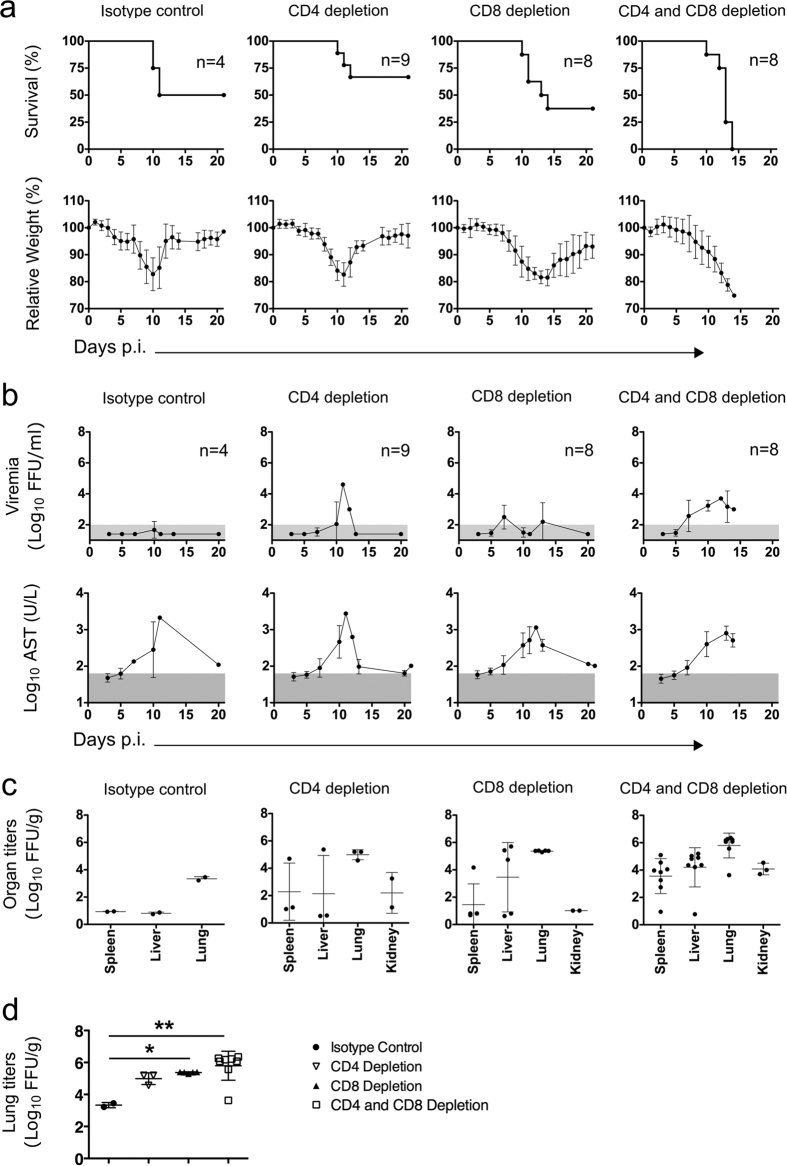
T cells are protective during EVD infection in WT → IFNAR^−/−^ chimeric mice. Chimeric WT → IFNAR^−/−^ mice were depleted of CD4 and/or CD8 T cells with anti-CD4 and/or anti-CD8 antibodies three days and one day before infection. Control mice received an Isotype control antibody. Depletion efficiency was analyzed via flow cytometry. Mice were infected i.n. with 1000 FFU of EBOV and survival and relative weight loss was measured (**a**). Viremia and AST activity were analyzed and organs titers were determined when mice were sacrificed due to termination criteria (**b**). The normal range for AST and the limit of detection for viremia in blood are shaded in grey. Mean and standard deviation are shown. Virus titers were determined in peripheral organs (**c**) as well as in lungs of infected mice. Statistics was assessed via Kruskal-Wallis test followed by Dunn’s post-test. ns (not significant) when *p* > 0.05, *(*p* ≤ 0.05), **(*p* ≤ 0.01) and ***(*p* ≤ 0.001) (**d**).

To deplete T cells *in vivo* we utilized intraperitoneal administration of monoclonal antibodies against CD8 and/or CD4 in WT → IFNAR^−/−^ chimeras and compared the effect of specific T cell depletion in these mice with those treated with isotype control antibody. Individual depletion of CD4 and CD8 T cells resulted in moderate increase of viremia, but did not significantly influence survival and morbidity. However, depletion of both CD4 and CD8 T cells completely abolished protection and resulted in uniformly lethal EVD (Fig. 4a). In addition, T cell depletion resulted in viremia and virus replication in peripheral organs (Fig. 4b and c). Furthermore, depletion of CD8 T cells alone or in combination with CD4 T cell depletion resulted in significant increase of EBOV replication in the lungs (Fig. 4d). These results indicated that T cell immunity was essentially required to control local EBOV replication and to prevent systemic virus dissemination.

## Discussion

Previous research has shown that EBOV infects DCs and macrophages derived *in vitro* from hematopoietic progenitors which was consistent with early studies showing the presence of EBOV virions in dendritic-like cells in tissue sections^3^,^4^,^5^,^6^. However, DCs derived *in vitro* with GM-CSF only mimic features of inflammatory monocyte-derived DCs but not of conventional Ftl-3-dependent DCs^22^,^23^. Thus, it is not possible to infer from these previous studies whether EBOV infects all DCs or only discrete subsets. Due to the fact that DC subsets have non-overlapping functions, this question is of key importance to understand the pathophysiology of EVD. Here we describe the kinetics of EBOV infection *in vivo* after mucosal exposure, a presumed natural route of infection. We show that the only populations infected in the respiratory mucosa were alveolar macrophages, conventional CD11b^+^ DCs and inflammatory moDCs. Surface EBOV GP staining was not detected in monocytes and neutrophils *in vivo*, in agreement with these cells being refractory and non-productively infected respectively^19^,^24^. We also failed to detect EBOV infection in any other cell subset, including non-hematopoietic CD45^−^ cells as well as in T and B lymphocytes. While it is known that T cells are spared from infection, other cell types including fibroblasts, endothelial and epithelial cells have been described as EBOV targets^2^. Indeed, since virus titers grow steadily in the lung for several days before viremia, there must be one or several cell types that support initial EBOV replication. These could be resident antigen-presenting cells such as CD11b^+^ DCs and macrophages, or other cells where the infection levels are below our detection limit. Additional research technology, which may be difficult to implement in BSL4 containment (i.e. individual cell sorting), may be necessary to address the early kinetics of EBOV infection *in vivo*.

The fact that inflammatory moDCs but not monocytes supported productive viral replication is noteworthy and points out to an important role of inflammation for EBOV infection and pathogenesis. Our results support the finding that while EBOV attaches to the surface of monocytes it can only enter these cells during their differentiation to moDCs which depends on the inflammatory environment^19^. However, abrogation of monocyte entry into the infected tissue in the CCR2^−/−^ model did not prevent virus dissemination and disease, which indicated that despite moDCs being preferred early EBOV targets, ferrying of infectious viruses by other migratory populations such as conventional CD11b^+^ DCs, or draining of free virus through the lymphatic system, may be sufficient for viral spread from the infection site to the body. Interestingly, one of the main migratory DC subsets, the CD103^+^ DCs does not seem to play a role on EBOV dissemination since we did not detect expression of viral GP in the surface of CD103^+^ DCs. Interestingly, there are substantial parallelisms between our study and a recent HIV report which indicated that productive infection of DC-SIGN^+^ but not langerin^+^ DCs by HIV depended on the C-type lectin receptor usage^25^. Since CD103^+^ DCs express langerin^26^ but not DC-SIGN, it seems worthwhile to determine whether EBOV can actually infect CD103^+^ DCs or rather whether the virus cannot replicate in these cells. CD103^+^ DCs are essential to cross-prime CD8 T cells, and thus, it would be important to assess the functionality of these cells *in vivo* both in animal models and the CD141^+^ human equivalent^27^.

Our findings point out at CD11b^+^ DCs (bona-fide and monocyte-derived) as putative vessels for EBOV dissemination from the initial point of entry and replication to the draining lymph nodes. This hypothesis is substantiated by the mobility of these cells and previous studies showing their capacity to transport pathogens to the lymphoid tissues^11^,^16^,^19^. We cannot rule out however, that other mechanisms such as drainage of free virus through the lymphatic system may be responsible, at least to some extent, for EBOV dissemination.

Many studies have shown that, in particular in the mouse model, susceptibility is associated to a great extent to the capacity of the mice to mount IFN-I dependent antiviral responses^9^. Consistently, IFNAR^−/−^ → IFNAR^−/−^ control chimeras reproduced the 100% lethality observed in IFNAR^−/−^ infected with non-adapted EBOV^9^,^12^. IFNAR^−/−^ → IFNAR^−/−^ control chimeras also displayed significantly higher levels of early virus replication in the lung than their WT → IFNAR^−/−^ counterparts, suggesting that IFN-I-dependent control of early EBOV replication is a key determinant of disease outcome. However, strikingly the pattern of EBOV infection did not depend on IFN-I competence. This was different from previous studies with, for example, influenza virus where the capacity of DCs to respond to IFN-I was determinant for infection of these cells *in vivo*^28^. These findings suggest that the susceptibility of IFNAR^−/−^ and STAT1^−/−^ mice to EBOV depends not only on the effects of IFN-I the coordination of in innate immune responses^12^,^29^, but also on the effect of this cytokine in adaptive immunity. This is also consistent with our observation that non-adapted EBOV replicated in the lungs of WT mice. It is conceivable that attachment factors such as C-type lectin receptors may determine EBOV tropism after which the action of virus-encoded IFN-I antagonists may be sufficient to ensure virus replication in target cells^29^,^30^,^31^.

In line with the importance of adaptive immunity for EBOV pathogenesis, we demonstrated that T cells are chief controllers of EBOV dissemination. As demonstrated before in other model systems including NHPs, both CD4 and CD8 T cells were required for protection in agreement with the idea that EBOV survival requires both arms of the adaptive immune response, namely, cytotoxic T cells and antiviral antibodies^2^,^3^,^4^. The importance of T cell function for protection is also consistent with recent findings from our group which indicated that fatal EVD was related with dysregulation of T cell homeostasis in humans^32^. Our results point out at T cell immunity as an important correlate of protection against EVD, and suggest that harnessing of T cell function via immunotherapy merits further investigation.

## Methods

### Mice and bone marrow chimeras

IFNAR^−/−^ mice (C57Bl/6 background) were obtained from the Friedrich Loeffler Institute, Isle of Riems, Germany and bred in the animal facility of the Bernhard Nocht Institute for Tropical Medicine. CD45.1^+^ congenic C57Bl/6-Ly5.1 and CCR2^−/−^ mice (C57Bl/6 background) were purchased from Jackson Laboratories and bred in the animal facility of the Heinrich Pette Institute. Bone marrow chimeras were generated at the Heinrich Pette Institute. Four to eight weeks old female recipient mice were irradiated with a lethal dose (2 × 7 Gray, 2 hr apart) in a Cs^137^ irradiator and then transplanted with 3 × 10^6^ bone marrow cells from donor mice. Engraftment of donor cells was analyzed in peripheral blood four weeks post-transplantation. Bone marrow chimeric mice with an engraftment of 85% and higher were utilized for the experiments.

### Experimental EBOV infection

This study was carried out in strict accordance with the recommendations of the German Society for Laboratory Animal Science and under the supervision of a veterinarian. The protocol was approved by the Committee on the Ethics of Animal Experiments of the City of Hamburg (permit no. 125/12). All efforts were made to minimize the number of animals used for experiments and to alleviate suffering of animals during experimental procedures. All staff carrying out animal experiments passed an education and training program according to category B or C of the Federation of European Laboratory Animal Science Associations. All experimental infections described in this study were performed within the biosafety level 4 (BSL4) facility at the Bernhard Nocht Institute for Tropical Medicine in Hamburg in accordance with institutional safety guidelines. Personnel wore appropriate protective equipment (biosafety suits). Ebola virus H.sapiens-tc/COD/1976/Yambuku-Mayinga was used for all infection experiments. Mice were anesthetized with isoflurane and infected intranasally (i.n.) with 1000 focus forming units (FFU) of EBOV in 50 μl PBS and monitored daily for signs of disease. Body weight and rectal body temperature were measured daily and blood (30–50 μl) was drawn via the tail vein every 2–7 days over a period of three weeks for clinical chemistry and viremia. Animals with severe symptoms such as temperature <28 °C or weight loss >20% were euthanized with isoflurane anesthesia followed by cervical dislocation.

### T cell depletion

CD4 and CD8 T cells were depleted via intraperitoneal administration of anti-CD4 (YTS191, BioXcell) and anti-CD8 (YTS169.4, BioXcell) depleting antibodies three and one day prior to infection (total of 300 μg per application). A control group received an isotype control antibody (LFT-2, BioXcell) at the same time and dose.

### Flow cytometry

For flow cytometry analysis lungs of infected animals were harvested and digested with Collagenase D (2 mg/ml, Roche) and DNAseI (50 μg/ml, Sigma). Single cell suspensions were treated with BD Pharm Lysing Buffer (BD Bioscience); cells were blocked with CD16/CD32 Fc-Block antibody followed by staining with an antibody cocktail. All fluorochrome-conjugated antibodies were purchased from BD Biosciences, eBiosciences or BioLegend. Monoclonal antibodies against EBOV GP (5D2 and 5E6) have been previously described^14^. A mix of equal parts 5D2 and 5E6 was conjugated with Alexa Fluor 488 using an antibody labeling kit (life technologies). Formaldehyde inactivated samples were aquired using a LSRFortessa (BD Bioscience) and analysed with FlowJo Software (FlowJo, LLC).

### Virus Titrations

Organs were homogenized in 1 ml of DMEM containing 2% FCS using Lysing Matrix D tubes (MP Biomedical) and a FastPrep homogenizer. To quantify infectious particles in supernatants of homogenized organs and blood samples a focus formation assay was utilized as described elsewhere.

### Clinical chemistry

To determine the levels of aspartate aminotransferases (AST) serum was diluted 1:10 or higher with a 0.9% saline solution and measured with a commercial kit from Roche and a Reflotron. The normal range for mice was determined in 20 unifected mice and was 40–60 U/l.

### Statistical analysis

Statistical analyses were done using Graphpad Prism 5 software. Differences in organ titers and infection rates of immune cells were analyzed using non-parametric statistics (Kruskal-Wallis test followed by Dunn’s post-test). The levels of significance were represented as follows: ns (not significant) when *p* > 0.05, *(*p* ≤ 0.05), **(*p* ≤ 0.01) and ***(*p* ≤ 0.001).

### Immunofluorescence analysis

Tissues were fixed in 4% buffered formalin and processed for paraffin embedding. For the detection of viral protein, mounted sections (2 μm) were dewaxed and antigen retrieval was performed for 30 min at 96 °C in 10 mM citrate buffer pH 6.0. Sections were washed once and blocked in blocking buffer (Protein-Free T20 (TBS) Blocking buffer #37071 Thermo Fischer) for 1 h. Anti-Ebola antibodies (5E6 and 5D2) and respective isotype control antibody (mouse IgG2) were both conjugated to Alexa488 according to the manufacturer’s protocol (Molecular Probes). Directly conjugated EBOV-Alexa488 or isotype-control-Alexa488 antibody (1:40 in blocking buffer each), respectively, was applied on the sections over 3 nights at 4 °C with gentle agitation. Afterward, sections were intensively washed and anti-CD11b antibody (1:2,000 in blocking buffer; #ab133357 Abcam) was applied overnight at 4 °C. Sections were washed again, followed by incubation with Alexa555-conjugated secondary anti-rabbit antibody for 1.5 h at room temperature. After repeated washing, sections were mounted with DAPI-Fluoromount-G (SouthernBiotech, Birmingham, USA). Data acquisition was performed using a Leica Sp5 confocal microscope and Leica application suite software (LAS-AF-lite).

## Additional Information

**How to cite this article:** Lüdtke, A. *et al*. Ebola virus infection kinetics in chimeric mice reveal a key role of T cells as barriers for virus dissemination. *Sci. Rep.* **7**, 43776; doi: 10.1038/srep43776 (2017).

**Publisher's note:** Springer Nature remains neutral with regard to jurisdictional claims in published maps and institutional affiliations.

## Supplementary Material

Supplementary Information
