# Supplementary material for: Ebola virus infection kinetics in chimeric mice reveal a key role of T cells as barriers for virus dissemination
Source: Sci Rep. 2017 Mar 3;7:43776. doi: 10.1038/srep43776 (PMC5335601; doi:10.1038/srep43776)
Supplement: Supplementary Information [file srep43776-s1.pdf]

Supplementary Information

Ebola virus infection kinetics in chimeric mice reveal a key role of T cells as barriers for virus dissemination

Anja Lüdtke,[1,2,3] Paula Ruibal,[1,2,3] David M. Wozniak,[2,3] Elisa Pallasch,[2,3] Stephanie Wurr,[2,3] Sabrina Bockholt,[2,3] Sergio Gómez-Medina,[1] Xiangguo Qiu,[4] Gary P. Kobinger,[4] Estefanía Rodríguez,[1] Stephan Günther,[2,3] Susanne Krasemann,[5] Juliana Idoyaga,[6] Lisa Oestereich[2,3] and César Muñoz-Fontela[1,2,3].

[1]Heinrich Pette Institute, Leibniz Institute For Experimental Virology, Martinistrasse 52 20251 Hamburg, Germany.

[2]Department of Virology, Bernhard Nocht Institute for Tropical Medicine, 20359 Hamburg, Germany

[3]German Center for Infection Research (DZIF), Partner site Hamburg.

[4]Special Pathogens Program, National Microbiology Laboratory, Public Health Agency of Canada, Winnipeg, MB R3E 3R2, Canada.

[5]Institute for Neuropathology, University Medical Center Hamburg-Eppendorf, 20251 Hamburg, Germany

[6]Department of Microbiology and Immunology, Stanford University School of Medicine, Stanford, CA 94305 USA.

SUPPLEMENTARY FIGURE LEGENDS

Supplementary Figure S1. Gating strategy for lung myeloid populations. Lung myeloid populations were gated as follows: G1 and G2: live cells, G3: singlet cells, G4: CD45 positive hematopoietic cells, G5: Ly6G negative cells, G6: neutrophils, G7: SiglecF negative cells, G8: SiglecF positive cells, G9: macrophages, G10: CD3 and B220 negative cells, G11: monocytes, G12: MHCII positive antigen-presenting cells, G13: Ly6C low monocytes, G14: Ly6C high monocytes, G15: activated monocytes, G16: DCs, G17: CD103 positive DCs, G18: CD11b positive DCs (A). Discrimination between bona-fide $CD11b^{+}$ conventional DCs and monocyte-derived DCs based on Mar-1 and CD64 staining (B).

Supplementary Figure S2. EBOV infection of additional cell subsets in the lung. Infection kinetics of stromal $CD45^{-}$ cells in WT→$IFNAR^{-/-}$ and $IFNAR^{-/-}$→$IFNAR^{-/-}$ chimeras. Fluorescence minus one (FMO) and Mock-infected controls are shown (A). Graph depicting infection rates of the indicated cell subsets in both chimeras over time (B).

Supplementary Figure S3. Frequency of infected DCs within all hematopoietic cells. Graph depicting the infection kinetics of bona-fide conventional $CD11b^{+}$ DCs ($coCD11b^{+}$ DCs) and monocyte-derived DCs ($moCD11b^{+}$ DCs) over time in the lung of $IFNAR^{-/-}$ mice. Numbers represent percentage of infected cells on each subset within all $CD45^{+}$ cells. Virus titer kinetics in the lung is also shown.

Supplementary Figure S4. EBOV infection of IFNAR$^{-/-}$ $^{CCR2-/-}$ chimeras. Four weeks post transplantation chimeric IFNAR$^{-/-}$ $^{CCR2-/-}$ mice were infected i.n. with 1000 FFU of EBOV and survival and relative weight loss was monitored over the course of infection (A). At indicated time points mice were bleed for viremia and AST activity, and euthanized due to termination criteria. Spleen, liver, lung and kidney were collected for viral titers. Statistical analysis was performed via non-parametric Kruskal-Wallis test followed by Dunn's post-test. ns (not significant) when $p > 0.05$, * ($p \leq 0.05$), ** ($p \leq 0.01$) and *** ($p \leq 0.001$) (B). The normal range for AST and the limit of detection for viremia in blood are shaded in grey. Graphs represent mean value ± SD.

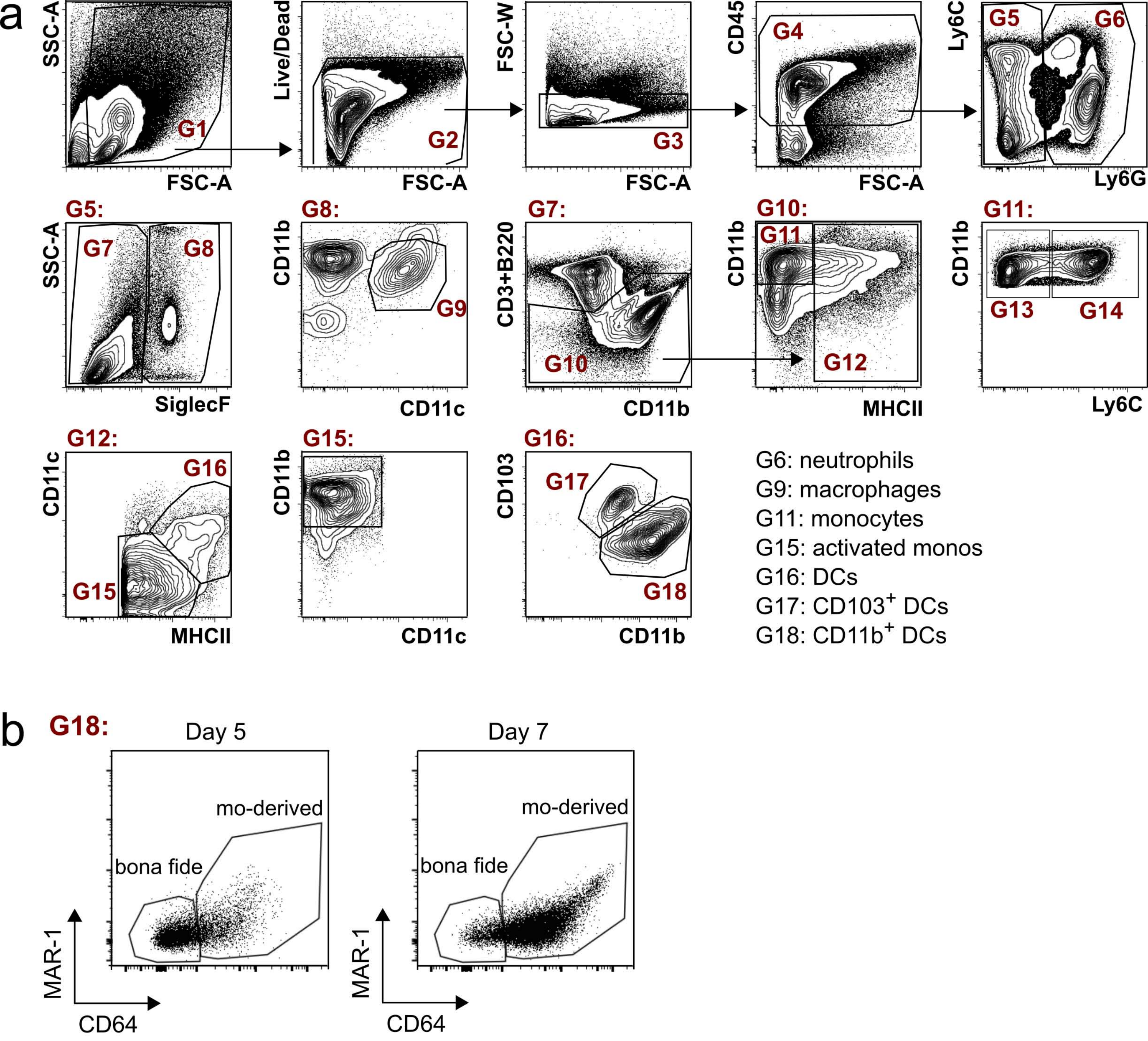
a
SSC-A
FSC-A
G1
Live/Dead
G2
FSC-W
G3
CD45
G4
Ly6C
Ly6G
G5
G6
G5:
SiglecF
G7
G8
G8:
CD11b
CD11c
G9
G7:
CD3+B220
CD11b
G10
G10:
MHCII
G11
G12
G11:
G13
G14
G12:
G16
G15
G15:
G16:
CD103
G17
G18
G6: neutrophils
G9: macrophages
G11: monocytes
G15: activated monos
G16: DCs
G17: CD103+ DCs
G18: CD11b+ DCs
b
G18:
Day 5
Day 7
mo-derived
bona fide
MAR-1
CD64

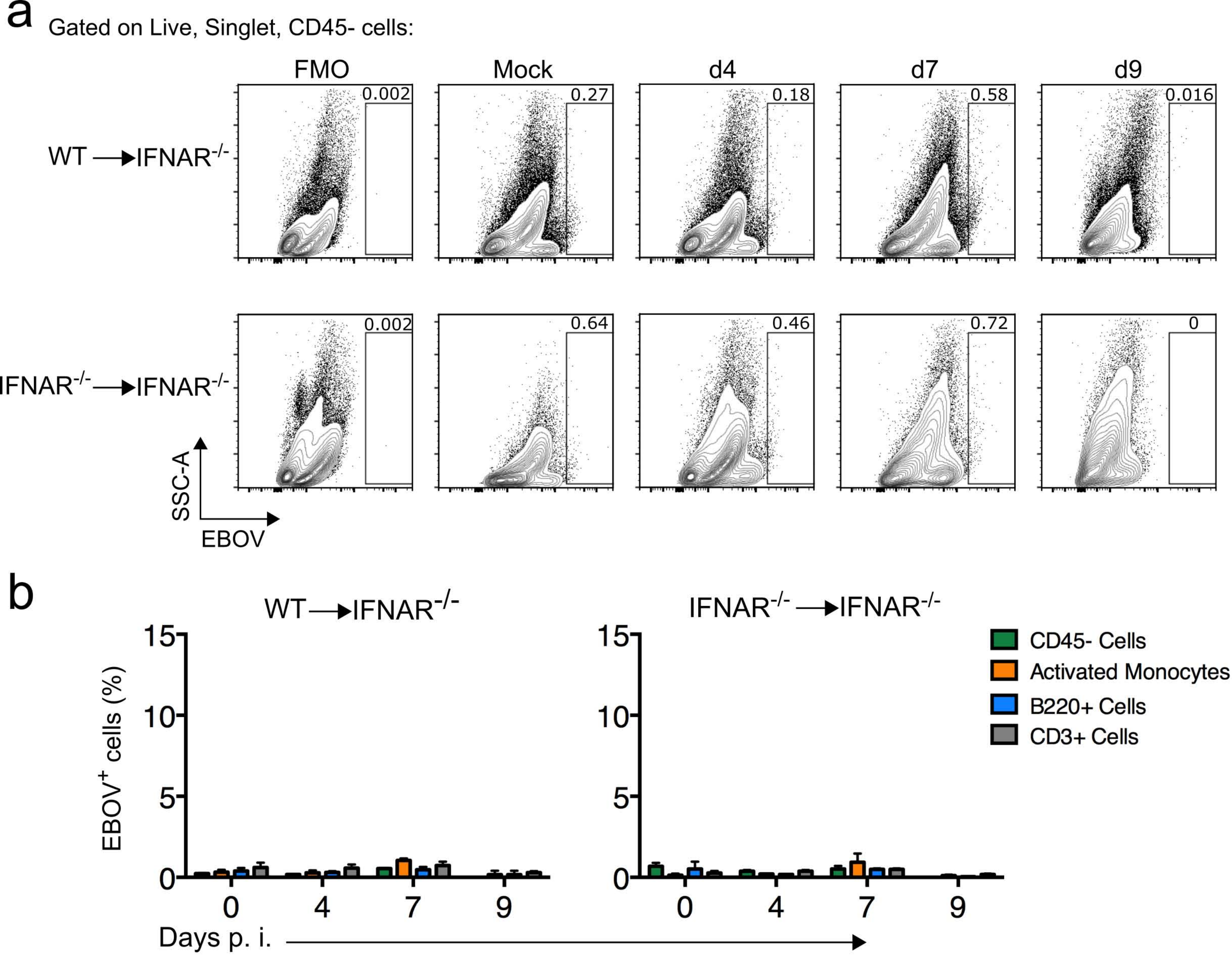
a
Gated on Live, Singlet, CD45- cells:
FMO
Mock
d4
d7
d9
WT → IFNAR-/-
0.002
0.27
0.18
0.58
0.016
IFNAR-/- → IFNAR-/-
0.002
0.64
0.46
0.72
0
SSC-A
EBOV
b
WT → IFNAR-/-
IFNAR-/- → IFNAR-/-
EBOV+ cells (%)
15
10
5
0
0
4
7
9
Days p. i.
CD45- Cells
Activated Monocytes
B220+ Cells
CD3+ Cells

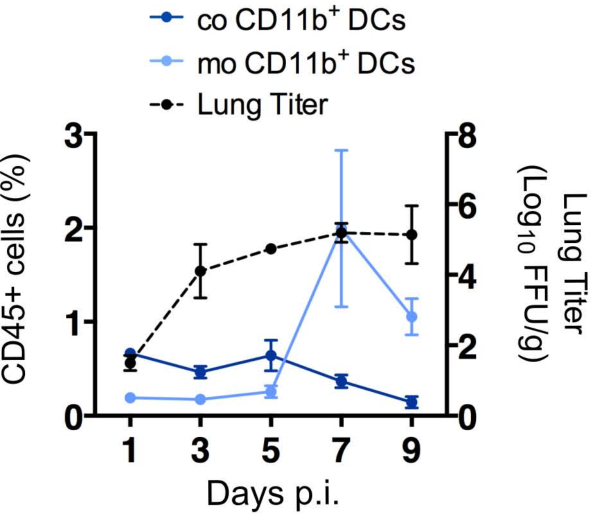

co CD11b+ DCs
mo CD11b+ DCs
Lung Titer
CD45+ cells (%)
3
2
1
0
8
6
4
2
0
Lung Titer (Log10 FFU/g)
1
3
5
7
9
Days p.i.

Suppl Fig 3

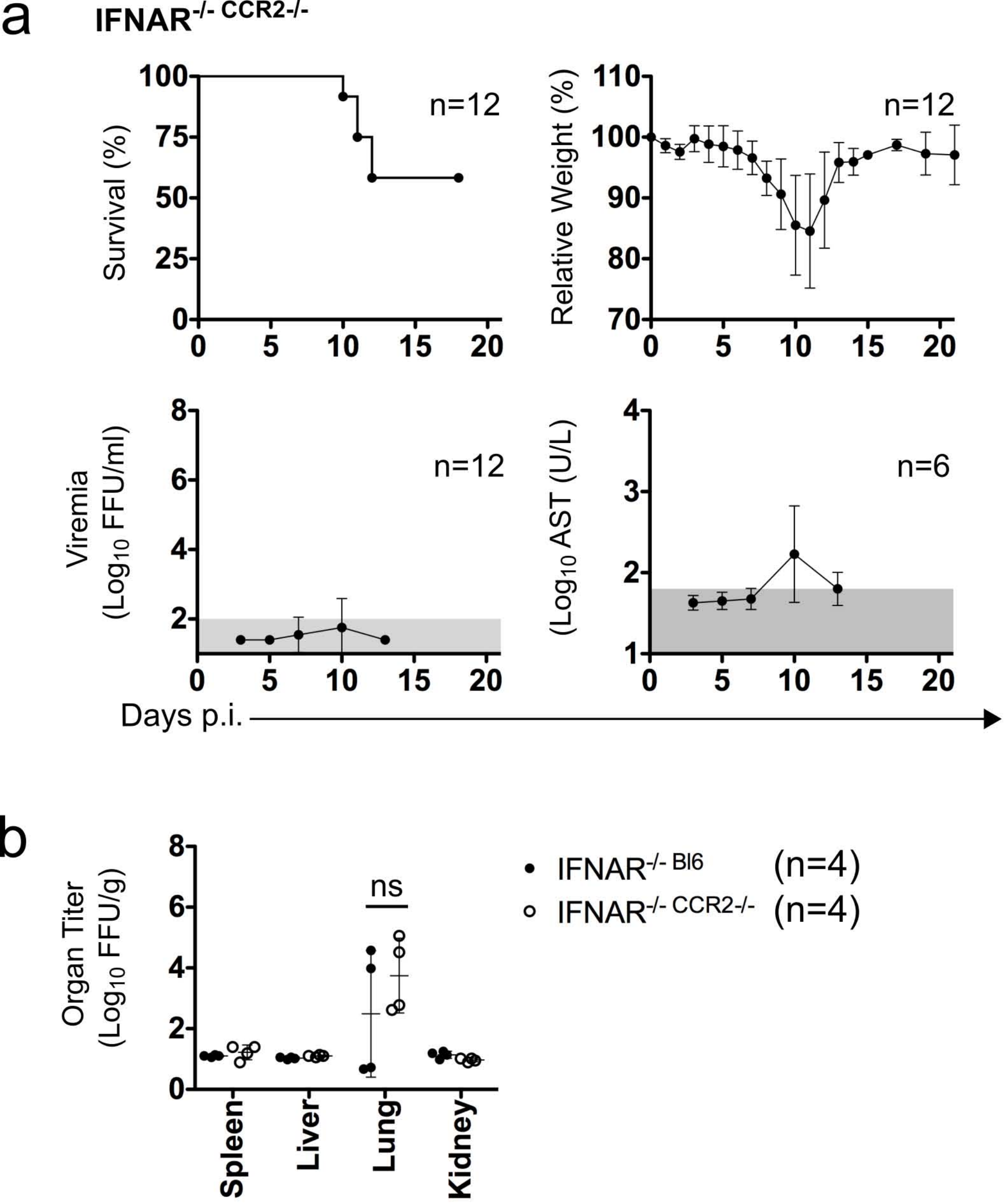
a
IFNAR-/- CCR2-/-
Survival (%)
n=12
Relative Weight (%)
n=12
Viremia (Log10 FFU/ml)
n=12
(Log10 AST (U/L)
n=6
Days p.i.
b
Organ Titer (Log10 FFU/g)
ns
Spleen
Liver
Lung
Kidney
IFNAR-/- Bl6 (n=4)
IFNAR-/- CCR2-/- (n=4)

Suppl Fig 4
